# Seroprevalence of transfusion-transmitted infections: A five-year multicentric study of 87,878 blood donors in the Mumbai region, India

**DOI:** 10.1016/j.htct.2026.106492

**Published:** 2026-07-10

**Authors:** Yoganand Vishwasrao Patil, Mrunal Vijay Kesari, Kalaivanan V, Shilpa Jain, Dina Abhani, Sagarika Khobaragade

**Affiliations:** a Jagjivan Ram Hospital, Western Railway, Mumbai, India; b Blood centre - Jagjivan Ram Hospital, Western Railway, Mumbai, India; c Bloodline Charitable Blood Bank, Thane, India; d Sadguru Charitable Blood Bank, Navi Mumbai, India

**Keywords:** Transfusion-transmitted infections, Seroprevalence, Blood donors, Blood safety, Hemovigilance

## Abstract

**Introduction:**

Blood transfusion is a critical component of modern medicine, yet the risk of transfusion-transmitted infections remains a significant challenge. Despite stringent screening protocols, infections such as hepatitis B, hepatitis C, human immunodeficiency virus, and syphilis continue to pose risks. Understanding the seroprevalence of transfusion-transmitted infections in blood donors is essential for improving transfusion safety and shaping public health strategies. The objective of this study is to evaluate transfusion-transmitted infection seroprevalence, analyze trends, and assess seropositivity by age, donation type, and co-infections over five years.

**Materials and methods:**

A five-year cross-sectional study (2020–2024) was conducted at three blood centers: Jagjivan Ram Hospital Blood Bank (Mumbai), Bloodline Charitable Blood Bank (Thane), and Sadguru Charitable Blood Centre (Navi Mumbai). The study assessed the prevalence of transfusion-transmitted infections by screening 87,878 donor samples for human immunodeficiency virus, hepatitis B, hepatitis C, syphilis, and malaria using standardized diagnostic methods. Donor demographics, infection patterns, and regional variations were analyzed statistically.

**Results:**

The overall seroprevalence of transfusion-transmitted infections was 0.78%, with Hepatitis B being the most common infection (0.48%), followed by syphilis (0.144%), human immunodeficiency virus (0.078%), and hepatitis C (0.075%). No malaria cases were detected. Seropositivity was highest among younger donors (18–30 years) and predominant among males (97%). Mumbai recorded the highest seroprevalence, followed by Navi Mumbai and Thane. Co-infections were rare, with human immunodeficiency virus with syphilis being the most frequently observed combination.

**Conclusion:**

This study identifies Hepatitis B as the most common transfusion-transmitted infection, followed by syphilis, human immunodeficiency virus, and hepatitis C. This study emphasizes the need for better detection strategies, specifically the use of enzyme-linked immunosorbent assay, electrochemiluminescence Immunoassay, and individual donor nucleic acid testing to improve blood safety. Transfusion-transmitted infection screening plays a vital role as a quality indicator for blood banks, driving method evaluation and improvements. Mumbai’s structured referral system ensures seropositive donors receive counseling and treatment, reinforcing blood safety protocols.

## Introduction

Approximately 118.54 million units of blood are collected globally each year, with 40% of the supply coming from high-income countries, which make up 16% of the world’s population [1]. Blood is often regarded as the essence of life, but when transfused, it carries both the potential to heal and the hidden risk of harm. Despite rigorous screening measures, Transfusion Transmitted Infections (TTIs), including Hepatitis B Virus (HBV), Hepatitis C Virus (HCV), Human Immunodeficiency Virus (HIV), and syphilis, continue to pose significant challenges to transfusion safety worldwide [2].

The seroprevalence of TTIs varies by region, shaped by donor demographics, socio-economic factors, and advancements in screening. Recognizing these patterns is key to improving blood safety and public health strategies.

Infected blood transfusions pose significant health risks, affecting not only recipients but also their communities. Ensuring transfusion safety involves improving donor selection, screening, and pathogen inactivation to minimize infections [3]. Optimal use of blood minimizes unnecessary transfusions, reduces infection risks, and conserves resources. Evidence-based practices and patient blood management enhance safety and ensure a sustainable blood supply.

Although more sensitive methods are available to detect TTI markers, false negatives still occur due to factors like asymptomatic carriers, donations during the window period, highly variable viral strains, and technical errors. As a result, preventing TTIs remains a significant challenge in transfusion medicine [4].

Hepatitis B remains the most common TTI, affecting 66% of the population in high-prevalence areas, with two billion infections and 400 million chronic carriers, causing 60% to 80% of primary liver cancers and 620,000 deaths annually [5,6]. Hepatitis C affects 3% of the global population, placing 170 million at risk, with India having 10–24 million active cases and a seroprevalence of 0.09% to 2.02% [7]. HIV, though declining, still had 1.7 million new cases in 2019, with India’s adult prevalence at 0.2% to 0.3% [8].

The objective of this study is to evaluate TTI seroprevalence, analyze trends, and assess seropositivity by age, donation type, and co-infections over five years. This study is novel in its comparative analysis of TTI prevalence across multiple blood centers using different serological methods, offering insights into detection effectiveness and regional variations.

## Materials and methods

A Multicentric cross-sectional study was conducted from January 1, 2020 to December 31, 2024, across three blood centers: the Blood bank, Jagjivan Ram Hospital (Western Railways, Mumbai), Bloodline Charitable Blood Bank (Thane), and Sadguru Charitable Blood Centre (Navi Mumbai). This study was conducted in Mumbai due to its dense population, cosmopolitan nature, and significance as a major healthcare hub, making it an ideal location to assess TTI prevalence in a diverse donor population. The western region of Mumbai includes Thane and Navi Mumbai, with one center selected from each zone.

Ethical approval was granted by the Institutional Review Board (IEC No EC/20/00156).

The inclusion criteria were blood donors aged 18–65 years, weighing at least 45 kg, and with a hemoglobin level of at least 12.5 g/dL. The upper age limit for first-time donors was 60 years, while repeat donors had an upper age limit of 65 years. These criteria were based on the Standard Operating Procedures of the respective blood centers, following the guidelines for blood donor selection and referral [9].

The exclusion criteria were individuals with high-risk behavior, asthmatics on steroids, individuals taking anticoagulants, and others as specified in the Standard Operating Procedures for blood donor selection and referral [9].

Healthy blood donors were evaluated by trained blood transfusion officers through a comprehensive medical history assessment and physical examination, adhering to the Blood Centre's standard operating procedures. Donors filled out a questionnaire in English, Marathi, or Hindi, providing personal information (name, age, gender, etc.) along with health details (general well-being, medical history, high-risk behaviors, etc.). After receiving counseling from a trained professional, eligible donors gave their consent and were assigned a unique identification number. Blood collection involved drawing 2 mL of venous blood for serological testing and 2 mL of blood in ethylenediaminetetraacetic acid (EDTA) for malaria testing with both procedures being performed under aseptic precautions.

## Serological analyses

At Jagjivan Ram Hospital Blood Center (Western Railways, Mumbai), transfusion-transmitted infections (TTIs) were screened using serological methods. Separate serum samples were tested for HIV, HBV, HCV, syphilis, and malaria. HIV detection was performed using 4th generation Qualisa™ HIV Ag and Ab ELISA kits (Tulip Diagnostics Pvt. Ltd.), while HBsAg and HCV were detected using 3rd generation Qualisa™ ELISA kits (Tulip Diagnostics Pvt. Ltd.). Syphilis was diagnosed using the Carbogen Kit (Tulip Diagnostics Pvt. Ltd.), which is based on the Rapid Plasma Reagin (RPR) method and therefore represents a non-treponemal test. Malaria screening involved a peripheral smear examination with conventional microscopy and Rapid Diagnostic Tests (RDTs) using Bioline malaria antigen P.f HRP-II for *Plasmodium falciparum* and p-LDH for *Plasmodium vivax*.

At the Bloodline Charitable Blood Bank (Thane), serological screening was first performed using ELISA for HIV, HBV, and HCV, and the Carbogen kit (RPR method, Tulip Diagnostics Pvt. Ltd.) for syphilis. For samples that tested reactive by ELISA, additional confirmatory testing was performed using individual donor Nucleic Acid Testing (ID-NAT) for HIV, HBV, and HCV, in accordance with the national guidelines, the Drugs and Cosmetics Act, and under the regulatory framework of the Drugs Controller General of India (DCGI).

At Sadguru Charitable Blood Centre (Navi Mumbai), electrochemiluminescence immunoassay (ECLIA) was used instead of ELISA for detecting HIV, HBV, and HCV, while syphilis screening was performed using the Carbogen kit (RPR method, Tulip Diagnostics Pvt. Ltd.), in alignment with their testing protocol.

All three participating blood centers (Mumbai, Thane, and Navi Mumbai) used the Carbogen kit (Tulip Diagnostics), which is based on the Rapid Plasma Reagin (RPR) method, a non-treponemal test for syphilis screening. No treponemal assays (such as TPHA or FTA-ABS) were used in this study.

NAT in India is regulated under the Drugs and Cosmetics Act, 1940 & Rules 1945, and its adoption has been encouraged by the National Blood Transfusion Council (NBTC) and the National AIDS Control Organisation (NACO) guidelines of 2017. However, NAT is not mandatory in India; it remains optional, subject to availability of infrastructure, resources, and licensing. In the present study, NAT was performed only at the Bloodline Charitable Blood Bank, Thane, because this center had the required infrastructure and regulatory approval to implement individual donor NAT in addition to conventional serological screening. At the Jagjivan Ram Hospital (Mumbai) and Sadguru Charitable Blood Centre (Navi Mumbai), NAT was not adopted due to resource constraints and reliance on ELISA or ECLIA-based serological screening methods, which are still considered standard and permissible under Indian regulations.

Seropositive samples were first repeated in duplicate using the same screening assay (ELISA/ECLIA/Carbogen-RPR). A sample was considered reactive only if at least two out of three results were positive. At the Bloodline Charitable Blood Bank (Thane), samples that were initially reactive for HIV, HBV, or HCV on ELISA were further confirmed by individual donor NAT. For syphilis, only the Carbogen kit was used; donors with repeatedly reactive results were referred to government-approved reference laboratories for confirmatory treponemal testing. In case of discrepant results, donors were deferred and not included in the final seropositive count. All confirmed reactive donors were counseled confidentially, and their blood bags were discarded in accordance with biomedical waste management regulations.

Data collected were entered into Microsoft Excel Spreadsheet for Analysis.

## Results

Over a five-year period, a total of 87,878 blood donors were screened, of which 689 were found to be seropositive, yielding an overall seroprevalence rate of 0.78%. The annual seroprevalence rates for the years 2020, 2021, 2022, 2023, and 2024 were 0.93%, 0.59%, 0.67%, 0.89%, and 0.84%, respectively. The breakdown of seropositive cases for each infection type revealed that 69 (0.078%) donors were positive for HIV, 427 (0.48%) for HBV, 66 (0.075%) for HCV, and 127 (0.144%) for syphilis, with no cases of malaria being detected during the study period. Over time, there was a noticeable decline in the seroprevalence of HIV, HBV, and HCV, while the number of syphilis cases initially increased before gradually decreasing. Hepatitis B remained the most common infection among donors over the years, followed by syphilis, HCV, and HIV (Table 1).

**Table 1:** Total seroprevalence in five years.

Year	No. of donors	No. of seropositive donors (n)						Sero (+) (%)
		HIV	HBV	HCV	Syphilis	Malaria	Total	
2020	12,699	9 (0.07%)	89 (0.7%)	5 (0.03%)	16 (0.13%)	0	119	0.93%
2021	17,294	14 (0.08%)	65 (0.3%)	8 (0.04%)	16 (0.17%)	0	103	0.59%
2022	18,844	16 (0.08%)	82 (0.4%)	12 (0.06%)	17 (0.13%)	0	127	0.67%
2023	19,648	18 (0.19%)	93 (0.4%)	21 (0.1%)	44 (0.2%)	0	176	0.89%
2024	19,393	12 (0.14%)	98 (0.5%)	20 (0.1%)	34 (0.1%)	0	164	0.84%
Total	87,878	69 (0.078%)	427 (0.48%)	66 (0.075%)	127 (0.144%)	0	689	0.78%

The majority of seropositive donors were male (97%), with only a small fraction (3%) of the total seropositive cases found among females. This trend is consistent across all infections, with males showing a significantly higher seroprevalence for HIV, HBV, HCV, and syphilis (Table 2).

**Table 2:** Prevalence of different transfusion-transmitted infections among males and females.

Sex	No. of seropositive donors (n)					
	HIV	HBV	HCV	Syphilis	Malaria	Total
Male	69	415	63	122	0	669 (97%)
Female	0	12	3	5	0	20 (3%)
Total	69	427	66	127	0	689

The highest number of seropositive cases was observed in the 18–30 age group, with 279 cases (40.49%), while the lowest percentage was seen in those over 60 years, with just two cases (0.29%) (Table 3).

**Table 3:** Age wise distribution of transfusion-transmitted infections.

Age Group (Years)	No. of seropositive donors (n)					
	HIV	HBV	HCV	Syphilis	Malaria	Total
18–30	29	154	32	64	0	279 (40.49%)
31–40	24	147	19	33	0	223 (32.36%)
41–50	13	107	9	27	0	156 (22.64%)
51–60	3	16	6	3	0	28 (4.06%)
Above 60	0	2	0	0	0	2 (0.29%)

Based on the regional distribution of TTIs, Mumbai exhibited the highest seroprevalence (0.97%), followed by Navi Mumbai (0.91%) and Thane (0.63%). Although Thane had the largest absolute number of seropositive donors, its adjusted prevalence was the lowest, these figures indicate regional variations, with Thane showing the most significant number of seropositive cases, especially for HBV (Table 4).

**Table 4:** Region wise distribution of transfusion-transmitted infections.

Region	Total Donations	No. of seropositive donors (n)						
		HIV	HBV	HCV	Syphilis	Malaria	Total TTI positive cases	Prevalence (%)
Mumbai	27,520	36	144	21	66	0	267	0.97%
Thane	44,782	23	199	15	44	0	281	0.63%
Navi Mumbai	15,576	10	84	30	17	0	141	0.91%

Co-infection of HIV with other TTIs was observed in a few cases. Notably, there were five instances of HIV and syphilis co-infection, with four occurring in 2024. Additionally, one donor in 2024 was found to be co-infected with HIV, HBV, and HCV. All co-infected individuals were male, with the majority of cases being reported in the blood bank of Jagjivan Ram Hospital. These co-infections underscore the complexity of TTIs in blood donors, especially in relation to HIV (Table 5).

**Table 5:** Co-infection of seropositive donors.

No	Co-Infection	Age	Sex	Year	Region
1.	HIV And Syphilis	22	Male	2022	Mumbai
2.	HIV And Syphilis	47	Male	2024	Mumbai
3.	HIV And Syphilis	24	Male	2024	Mumbai
4.	HIV, HBV and HCV	47	Male	2024	Mumbai
5.	HIV And Syphilis	42	Male	2024	Mumbai

## Discussion

Each blood transfusion carries a 1% risk of transfusion-related reactions and the potential for TTIs [10]. Developed countries have significantly reduced TTIs in the past two decades through effective prevention efforts, while developing nations still struggle with early-stage, hospital-based blood transfusion policies [11]. Blood transfusions should follow national guidelines, considering the needs of the patient, minimizing costs and waste, ensuring safety, and maintaining effectiveness [12]. Given the long-term consequences of TTIs on recipients, healthcare organizations are responsible for ensuring safe blood transfusion services [13]. Continuous improvements in donor screening, selection, and testing methods are essential to reduce the risk of acquiring TTIs.

The total seroprevalence of TTIs in this study was 0.78%, which is low compared to other studies, such as Mendhe VV et al. (1.01%) [14], Lakshmikumar MT et al. (1.07%) [15], Patel SK et al. (1.19%) [16], and Bagde S et al. (1.46%) [17]; however, it is higher than the findings of Sharma RL et al. (0.36%) [18]] (Table 6). This lower prevalence may be attributed to stringent donor selection criteria, increased awareness among donors, effective pre-donation screening to exclude high-risk individuals, and a higher proportion of voluntary non-remunerated blood donors. Additionally, factors such as a predominantly young donor population, better socioeconomic conditions, higher literacy rates, and improved access to medical facilities contribute to reducing the overall burden of transfusion-transmitted infections.

**Table 6:** Regional variations in the seroprevalence of transfusion-transmitted infections across India.

Place	Publication Year	Total no of donors	Total seroprevalence %	HIV	HBV	HCV	Syphilis	Malaria
Karnataka Bommanahalli BP et al. [19]	2014	19,413	2.22%	-	2.12	0.1	-	-
Gurjat Dhruva GA et al. [20]	2014	10,788	0.93%	0.074	0.68	0.074	0.065	0.037
New Delhi Makroo RN et al. [21]	2015	180,477	11.9%	0.24	1.18	0.43	0.23	0
Western Himalayas Raina S et al. [2]	2015	27,995	0.83%	0.10	0.49	0.21	0.03	-
Gujrat Assessment of NACO supported blood banks- [22]	2016	606,683	1.11%	0.11	0.64	0.15	0.18	0.039
Gujrat Sharma RI et al. [18]	2018	13,724	0.36%	0.03	0.29	0	0.04	0
Andhra Pradesh Naik VSS et al. [23]	2020	54,937	2.41%	0.23	1.82	0.31	0.04	0.01
Haryana Cheema S et al. [24]	2022	10,797	1.07%	0.03	0.49	0.50	0.05	0.009
Maharashtra Mendhe VV et al. [14]	2023	12,193	1.01%	0.09	0.7	0.106	0.04	0
Gujrat Patel SK et al. [16]	2024	20,392	1.19%	0.09	0.76	0.14	0.20	0
**Present Study**	**2025**	**87,878**	**0.78%**	**0.078**	**0.48**	**0.075**	**0.144**	**0**

In the present study, the vast majority of donors (97%) were male, which is concordant with findings from studies by Rao and Annapurna, [25] Rose et al. [26] Arora D et al. [27] and Singh B et al. [28] all of whom reported male donors making up >90% of the donor population. The disparity in TTI seroprevalence between male and female donors may be influenced by male-specific risk factors, such as greater social exposure and polygamous relationships. Female donor underrepresentation is due not only to lower participation but also higher deferral rates, with many women being deferred for reasons like low hemoglobin levels and health-related factors. Additionally, limited awareness, lack of motivation, and insufficient education contribute to the lower female donor participation.

In this study, the highest prevalence of TTIs was observed in the 18–30 age group (40.49%), while the lowest was found in the over 60 age group (0.29%). This trend is concordant with studies by Mandal et al. [29] who reported peak prevalence in the 26–35 age group, and Mendhe et al. [14] who found the highest seropositivity in the 21–30 age group, with the lowest rates in those aged 50 and above. The higher TTI prevalence in the 18–29 age group may be due to greater social exposure, risk behavior, and first-time donations where infections are detected. In contrast, lower rates in donors above 60 years likely reflect reduced high-risk activities, cumulative screening, and safer practices among long-term voluntary donors.

All blood donors in this study were voluntary and unpaid. The predominance of voluntary donations underscores the significance of encouraging voluntary blood donation as a strategy to minimize the risk of transfusion-transmitted infections (TTIs), aligning with the objectives of India's National Blood Policy [30]. Additionally, the majority of repeat donors were voluntary and underwent regular testing, which likely contributed to the lower TTI rates observed in this group.

Hepatitis B emerged as the most prevalent transfusion-transmitted infection (TTI) in the current study, with a seroprevalence of 0.48%. This finding is concordant with similar studies, such as Cheema S et al. (HBV = 0.49%) [24], Mendhe VV et al. (HBV = 0.7%) [14], and Bhawani Y et al. (HBV = 1.41%) [31] that reported comparable rates of HBV prevalence among blood donors. The consistent occurrence of hepatitis B across multiple studies highlights the continued relevance of this infection in the context of transfusion safety. While blood banks implement screening procedures, the persistent prevalence of HBV underscores the need for enhanced donor education, vaccination programs, and the continued advancement of screening technologies. Additionally, it is important to consider the region-specific variations in prevalence, as they may reflect local epidemiological patterns and stress the importance of targeted interventions based on local prevalence data.

It is crucial to detect Hepatitis B during the window period; the implementation of NAT could potentially reduce the risk. However, the high cost of NAT limits its widespread use in many centers [32].

In this study, syphilis was identified as the second most prevalent transfusion-transmitted infection (TTI), with a seroprevalence of 0.144%, followed by HIV at 0.078% and HCV at 0.075%. These results are concordant with those observed in similar studies conducted by Patel SK et al. [16] and Sharma RI et al. [18] which reported comparable rates of syphilis, HIV, and HCV among blood donors.

No cases of malaria were detected among the donors in this study, which is concordant with the findings of other studies, including those by Kaur G et al. [33] Makroo RN et al. [21] and Sethi B et al. [34] where none of the donors tested positive for malarial parasites.

Co-infection was observed in 5 (0.005%) of the seroreactive donors, which is lower than that reported by Kaur R et al. (co-infection = 1.2%) [35] and Kaur H et al. (co-infection = 4.04%) [36]. The discordance in co-infection rates may stem from differences in study populations, geographic regions, testing protocols, and time periods, as well as variations in sample size and representativeness. These factors can influence the detection and prevalence of co-infections across studies. Co-infections are significant because they affect the course of disease and the quality of life, and share common risk factors and transmission routes.

In the present study, regional analysis showed that Mumbai had the highest seroprevalence (0.97%), followed by Navi Mumbai (0.91%) and Thane (0.63%). Although Thane contributed the largest absolute number of seropositive donors, the adjusted prevalence was lowest when calculated against the total donor pool. Importantly, seroprevalence values in this study were derived from serological assays alone. NAT primarily adds value by identifying window-period infections that are seronegative but NAT-positive, thereby reducing residual transfusion risk. Since no NAT-only cases were detected during the study period, prevalence values remained unaffected. The observed regional variations are therefore more likely explained by differences in donor demographics, distribution of first-time versus repeat donors, and local epidemiological factors rather than by assay type alone.

These findings emphasize the need for targeted interventions and region-specific strategies to enhance blood safety, particularly in areas with higher TTI prevalence.

Donors identified with TTIs are directed to Integrated Counseling and Testing Centers for HIV and government-approved facilities for HBV, syphilis, and malaria, ensuring they receive proper medical evaluation and necessary treatment [37].

## Strengths of this study

The strength of this study lies in its large sample size, multicentric design, and diverse serological methods, enhancing the reliability and generalizability of findings. By incorporating data from multiple regions and using ELISA, ECLIA, and individual donor NAT, it provides a comprehensive analysis of TTI prevalence while enabling regional comparisons and assessing the effectiveness of different detection techniques.

## Limitations

The study is limited by its focus on the Mumbai-Thane-Navi Mumbai region, which may not fully represent broader or rural populations with different socio-economic factors. This regional scope may affect the generalizability of TTI prevalence across diverse settings.

## Conclusion

TTI screening serves as a crucial quality indicator for blood banks, facilitating method evaluation and enhancement. This study highlights Hepatitis B as the most common TTI, followed by syphilis, HIV and HCV. The evaluation of ELISA, ECLIA, and individual donor NAT emphasizes the importance of refining detection strategies to improve blood safety. Additionally, Mumbai’s structured referral system ensures seropositive donors receive necessary counseling and treatment thereby strengthening the effectiveness of blood safety protocols.

## Research involving human participants

This study was been conducted in accordance with ethical guidelines. (IEC No EC/20/00156).

## Informed consent

Informed consent was obtained from all participants included in the study.

## Data availability

The data that support the findings of this study are available from the corresponding author upon reasonable request.

## Conflicts of interest

No conflicts of interest to declare.
